# Use of mesoglycan in the acute phase of hemorrhoidal disease (the CHORMES study): study protocol for a double-blind, randomized controlled trial

**DOI:** 10.1186/s13063-024-08648-y

**Published:** 2024-12-02

**Authors:** Gaetano Gallo, Arcangelo Picciariello, Alberto Realis Luc, Antonella Salvatore, Angelo Di Vittori, Marcella Rinaldi, Mario Trompetto

**Affiliations:** 1 Department of Surgery, Sapienza University of Rome, Rome, Italy; 2 Department of Experimental Medicine, University of Salento, Lecce, Italy; 3 Department of Colorectal Surgery, Santa Rita Clinic, Vercelli, Italy; 4 Department of Surgery, Colorectal Surgery Center, University of Cagliari, Cagliari, Italy; 5 Division of General and Hepatobiliary Surgery, Department of Surgical Sciences, Dentistry, Gynaecology and Paediatrics, University of Verona, Verona, Italy; 6 Departement of Precision and Regenerative Medicine and Ionian Area, Aldo Moro” University of Bari, Bari, Italy

**Keywords:** Acute hemorrhoidal disease, Conservative treatment, External thrombosed hemorrhoids, Hemorrhoidal crises, Hemorrhoidal disease, Mesoglycan

## Abstract

**Background:**

Hemorrhoidal disease (HD) is associated with substantial economic burden and negative effects on health-related quality of life (HRQoL). The aCute HaemORrhoids treatment with MESoglycan (CHORMES) study aims to evaluate the effects of orally administered mesoglycan, a natural preparation of glycosaminoglycans with antithrombotic and profibrinolytic properties, as an acute treatment in patients with HD.

**Methods:**

CHORMES is a phase 2, double-blind, randomized controlled trial being conducted at two centers in Italy. Adults aged 18–75 years with Grade I–III HD according to Goligher classification or external thrombosed hemorrhoids, and a Hemorrhoidal Disease Symptom Score (HDSS) of ≥ 5, will be randomly allocated in a 1:1 ratio to mesoglycan or placebo and will be treated for 40 days (two capsules for the first 5 days and one capsule for the subsequent 35 days twice daily [after breakfast and dinner], equivalent to 200 mg in the first 5 days and 100 mg subsequently). Concomitant use of analgesics is permitted in both treatment groups. The trial aims to enroll 50 patients, with 25 patients in each treatment group. The primary objective of the trial is to evaluate the efficacy of mesoglycan in reducing symptoms of HD, assessed via change in HDSS from baseline (day 0) to day 40 in the intention-to-treat population. Secondary objectives include changes in HRQoL from baseline to day 40 using the Short Health Scale for Hemorrhoidal Disease, safety (adverse effects, physical assessments, vital signs and laboratory parameters in the safety population), fecal continence assessed using the Vaizey score, bleeding assessed using the Bleeding score, the amount and type of analgesic taken, and pain. Patient enrolment began on 11 December 2023, and trial completion is expected by December 2024.

**Discussion:**

The CHORMES trial will evaluate the efficacy and safety of mesoglycan, in addition to its impact on HRQoL, analgesic use and pain, in patients with HD. The results of the trial will assist clinicians in determining the most effective treatment for patients with HD.

**Trial registration:**

ClinicalTrials.gov NCT06101992. Prospectively registered on 26 October 2023 at https://clinicaltrials.gov/ct2/show/NCT06101992.

**Supplementary Information:**

The online version contains supplementary material available at 10.1186/s13063-024-08648-y.

## Administrative information

Note: the numbers in curly brackets in this protocol refer to SPIRIT checklist item numbers. The order of the items has been modified to group similar items (see http://www.equator-network.org/reporting-guidelines/spirit-2013-statement-defining-standard-protocol-items-for-clinical-trials/).

**Table:** 

Title {1}	Use of mesoglycan in the acute phase of hemorrhoidal disease (the CHORMES study): study protocol for a double-blind, randomized controlled trial
Trial registration {2a and 2b}.	ClinicalTrials.gov NCT06101992. Prospectively registered on 26 October 2023 at https://clinicaltrials.gov/ct2/show/NCT06101992.
Protocol version {3}	Version 2.0 – Date: 18 April 2023
Funding {4}	Neopharmed Gentili S.p.A., Milan, Italy
Author details {5a}	^1^Department of Surgery, Sapienza University of Rome, Rome, Italy ^2^Department of Experimental Medicine, University of Salento, Lecce, Italy ^3^Department of Colorectal Surgery, Santa Rita Clinic, Vercelli, Italy ^4^Department of Surgery, Colorectal Surgery Center, University of Cagliari, Cagliari, Italy ^5^Division of General and Hepatobiliary Surgery, Department of Surgical Sciences, Dentistry, Gynaecology and Paediatrics, University of Verona, Verona, Italy ^6^Departement of Precision and Regenerative Medicine and Ionian Area, “Aldo Moro” University of Bari, Bari, Italy
Name and contact information for the trial sponsor {5b}	Stefania Ghivarello 0039 3,386,692,341 0039 0289132292
Role of sponsor {5c}	Neopharmed Gentili has oversight of the study design, data collection, analysis and interpretation, as well as of drafting of the manuscript and the decision to submit the report for publication.

## Introduction

### Background and rationale {6a}

Hemorrhoidal disease (HD) is the most common proctological disease, with an estimated prevalence of 11–39%, peaking in individuals aged 45–65 years [1–3]. HD is associated with a substantial economic burden and can negatively affect health-related quality of life (HRQoL) [2, 4].

The most common presentation of HD is painless rectal bleeding occurring during or immediately after defecation [3, 5, 6]. Other symptoms, typically associated with prolapsed or large hemorrhoids, include rectal itching or swelling, a sensation of rectal fullness, fecal soiling, and pain [3, 5–7]. The Goligher classification system [8] is typically used to grade the severity of HD [3, 6]. This system is based on the presence and severity of prolapse, and ranges from Grade I (hemorrhoidal engorgement above the dentate line but no prolapse) to Grade IV (prolapsed hemorrhoids that cannot be reduced manually, possibly accompanied by inflammatory changes, mucosal atrophy, and ulceration). However, it is important to note that the Goligher classification system does not consider a wide range of symptoms, apart from bleeding in Grade I HD, special conditions such as pregnancy or inflammatory bowel disease, comorbidities or the presence of a single or circumferential prolapse, which may introduce bias and interobserver variability between physicians on assessing HD using the Goligher classification [9].

Effective treatments for Grade I–III internal hemorrhoids include diet and lifestyle modifications and medical treatments (e.g., topical glucocorticoids, vasoconstrictors, analgesics) and/or, in the event of failure, outpatient non-operative procedures such as rubber band ligation, sclerotherapy, and infrared coagulation [3, 5, 6, 10, 11]. In patients with thrombosed external hemorrhoids, topical vasodilators (e.g., nifedipine, glyceryl trinitrate) can relieve pain and reduce the size of the hemorrhoid [11]. If these treatments are ineffective, or in the event of high-grade and symptomatic hemorrhoids, surgical intervention is indicated [6, 10, 12]. Patients awaiting surgery can obtain relief through use of local or systemic anti-inflammatory agents [12].

Glycosaminoglycans (GAGs) are a heterogeneous family of polysaccharides that are essential components of the endothelium in blood vessel walls [13, 14]. As well as being key structural and functional components of connective tissues, GAGs have been shown to be involved in several biological functions, including embryonic development and regulation of cell signaling [13, 14]. Mesoglycan, which is extracted from the intestinal mucosa of pigs, is a natural preparation of GAGs that is rich in sulfur radicals with a strong negative electrical charge [14–16]. It consists of heparan sulfate (47.5%), dermatan sulfate (35.5%), chondroitin sulfate (8.5%), and slow-moving heparin (8.5%) [14–16], and is available in oral formulations or as a topically applied medical device (Prisma® Skin) [15, 17].

Mesoglycan exerts antithrombotic (activation of antithrombin III and heparin cofactor II) and profibrinolytic (stimulation of tissue plasminogen activator) activity by decreasing plasma fibrinogen concentrations without influencing prothrombin time, partial thromboplastin time, or other coagulation parameters [15, 16, 18–23]. Mesoglycan also promotes wound healing and has anti-inflammatory and anti-edematous effects [15, 16, 19, 22–25]. Mesoglycan has been shown to reduce thrombosis of mucocutaneous bridges after excisional hemorrhoidectomy in patients with Grade III–IV HD [16, 18], which may help avoid further surgery and reduce the risk of anal stenosis [26]. Additionally, the results of two preliminary Italian studies have suggested that mesoglycan effectively relieves symptoms (pain, itching, edema, inflammation, rectal bleeding) in patients with acute HD [27, 28].

While mesoglycan is widely considered to be useful for the treatment of acute HD (i.e., thrombosed external hemorrhoids) [11], the level of literature evidence in this patient population is not robust, and additional data are needed. As lifestyle changes (high water intake, diet rich in fiber, stool softeners) may provide meaningful improvements in symptoms in all phases of hemorrhoidal disease, a placebo-controlled trial design is appropriate. Therefore, the phase 2 aCute HaemORrhoids treatment with MESoglycan (CHORMES) trial is being conducted to assess the effects of mesoglycan as an acute treatment in patients with HD; herein, we describe the design of and rationale for the trial.

### Objectives {7}

The CHORMES trial aims to evaluate the efficacy and safety of mesoglycan versus placebo in patients with HD (Grade I–III or thrombosed external hemorrhoids). It will examine the effects of treatment on symptoms of HD and its impact on patient quality of life. Analgesic use during treatment and pain will also be assessed.

### Trial design {8}

In this phase 2 double-blind, randomized, controlled clinical trial, mesoglycan will be compared with placebo, with patients randomly allocated to either of these treatments in a 1:1 ratio. Patients will receive mesoglycan or placebo for 40 days, with assessment of effects at study site visits after 10, 20, and 40 days (± 2 days) of treatment. A follow-up assessment (for adverse events [AEs] only) at day 70 will also be performed via telephone.

## Methods: participants, interventions, and outcomes

### Study setting {9}

The trial is being conducted at two tertiary referral centers in Italy – Azienda Ospedaliero Universitaria Consorziale Policlinico, Bari, and Clinica Santa Rita di Vercelli, Presidio di Policlinico di Monza, Monza.

### Eligibility criteria {10}

#### Inclusion criteria

- Adult patients (aged 18–75 years).
- Diagnosis of Grade I–III HD (according to the Goligher classification) or external thrombosed hemorrhoids.
- Hemorrhoidal Disease Symptom Score (HDSS) of ≥ 5.
- Female patients of childbearing age must agree to the use of an effective contraception method throughout the trial.

#### Exclusion criteria

Excluded patients are those with a medical history of:

- Coagulopathy (e.g., hemophilia A or B),
- Heart disease (e.g., atrial fibrillation, myocardial infarction),
- Chronic inflammatory bowel disease,
- Previous perianal surgery or other proctological pathology in the acute phase,
- Anal or colorectal neoplasms in the previous 5 years.

Excluded patients are those:

- Who are carriers of diathesis/hemorrhagic diseases,
- Who are pregnant,
- Who have received radiotherapy within the last 3 years or an anticoagulant or antiaggregant therapy,
- With galactose intolerance, Lapp lactase deficiency, or glucose-galactose malabsorption,
- Who have a contraindication to treatment or known intolerance to mesoglycan (or any of its excipients), or hypersensitivity to heparin and heparinoids.

### Who will take informed consent? {26a}

All patients must provide signed informed consent. Informed consent is being sought, obtained, and documented (in writing) by the Investigator in compliance with applicable regulatory requirements, Good Clinical Practice (GCP), and the ethical principles originating from the Declaration of Helsinki.

### Additional consent provisions for collection and use of participant data and biological specimens {26b}

Prior to starting the trial, the information sheet, informed consent form (Supplementary File S1), and protocol were submitted to the ethics committee of the Azienda Ospedaliero Universitaria Consorziale Policlinico, Bari (the coordinating center) and subsequently to the Clinica Santa Rita, Vercelli for review and approval (approval number 0067698). The trial was also approved by the Italian Medicines Agency (Agenzia Italiana del Farmaco, AIFA).

Investigators are ensuring that the privacy of patients, including their personal identity and all personal clinical information, will be maintained at all times. Personal clinical information will be treated as confidential in line with European Union privacy regulations, with patients identified only by individual identification codes in the electronic case report form (e-CRF) and other documents submitted to the Investigator. Data on the e-CRF, including personal clinical information, will be verified by the Monitor (an employee of the Contract Research Organization [CRO] Clinical Trial Consulting), persons specifically authorized by the Sponsor or by competent authorities.

## Interventions

### Explanation for the choice of comparators {6b}

Given the aforementioned anti-edematous, antithrombotic, and profibrinolytic actions of mesoglycan [15, 16, 18–25] and preliminary documentation of its clinical activity in patients with acute HD [16, 27, 28], it has been hypothesized that mesoglycan will effectively relieve symptoms of Grade I–III HD or external thrombosed hemorrhoids and improve HRQoL and will be associated with a low incidence and severity of AEs compared with placebo. Placebo has been chosen as the comparator in the trial because this will provide the clearest assessment of the benefits of mesoglycan in patients with HD [11, 12], while the concomitant use of analgesics and stool softeners in both treatment groups will be recorded to allow for the comparison between groups.

### Intervention description {11a}

Mesoglycan and placebo will be provided in boxes containing blister packs of 15 hard capsules each. The study drug will be identified by a unique batch number and expiry date. Each mesoglycan capsule contains mesoglycan sodium salt 50 mg (including heparin sulfate 47.5%, dermatan sulfate 35.5%, chondroitin sulfate 8.5%, and heparin slow 8.5%) plus excipients (lactose monohydrate, maize starch, croscarmellose sodium, magnesium stearate, gelatin, titanium dioxide, and erythrosine). The placebo capsules contain excipients (lactose monohydrate, maize starch, croscarmellose sodium, and magnesium stearate) and no active ingredients. Patients will take two capsules of mesoglycan or placebo for the first 5 days then one capsule for the next 35 days, twice daily (after breakfast and dinner). The total daily dose of mesoglycan will, therefore, be 200 mg in the first 5 days and 100 mg in the subsequent 35 days.

### Criteria for discontinuing or modifying allocated interventions {11b}

Treatment with the study drug may be discontinued spontaneously by the patient at any time or by decision of the Investigator, as the patient’s clinical condition suggests. For patients who discontinue the trial, a complete final evaluation will be performed, with results reported in the e-CRF alongside the reasons for discontinuation. Grounds for early termination of treatment included withdrawal of informed consent, protocol violations, and occurrence of serious/unexpected AEs or changes in health status that would interfere with the assessment of the trial or make it inappropriate to continue.

### Strategies to improve adherence to interventions {11c}

At each assessment visit, patients will return all empty blister packs and unused trial drug to check for compliance; they will then be given a sealed package containing the next 10 or 20 days’ treatment with trial drug or placebo. Investigators will ensure accountability for all drugs delivered/returned by patients using a drug accountability form.

### Relevant concomitant care permitted or prohibited during the trial {11d}

Permitted concomitant medications include analgesics, stool softeners, fecal emollients, and any treatments taken for unrelated conditions that will not interfere with the study drug.

Drugs that may interfere with the evaluation of the study drug or alter the evaluation of efficacy parameters are not permitted as concomitant therapy. These include (but are not limited to) oral anticoagulation therapy (e.g., warfarin, acenocoumarol), novel oral anticoagulants (e.g., rivaroxaban, apixaban, and dabigatran), and anti-platelet agents (e.g., double-anti-aggregation or acetylsalicylic acid > 160 mg/day and ticlopidine).

### Provisions for post-trial care {30}

After the end of the trial (last visit of the last patient enrolled), any new serious adverse drug reactions or any new information about previously recorded AEs will be reported by the Investigators to the Sponsor. All AEs detected during the trial will be followed up until resolved, judged to be stable or no further information on the event/final outcome is possible.

### Outcomes {12}

#### Primary outcomes

The primary objective of the trial is to demonstrate the superiority of mesoglycan versus placebo in reducing symptoms of HD (i.e., the change in HDSS from baseline to day 40). The HDSS is a 5-item questionnaire that asks patients about HD symptoms experienced using a scale of 0 (never) to 4 (every day/always). Total score ranges from 0 to 20, with a higher score indicating more frequent and severe symptoms [29]. Symptoms of HD will be assessed using the HDSS at baseline (day 0; T0), day 10 (T1), day 20 (T2), and day 40 (T4).

#### Secondary outcomes

Secondary objectives of the trial are to assess (i) changes in HRQoL from baseline to day 40 using the Short Health Scale for Hemorrhoidal Disease (Short Health Scale_HD_); (ii) safety (AEs, physical assessments, vital signs and laboratory parameters); (iii) fecal continence (using the Vaizey score); (iv) bleeding (assessed via study-defined bleeding score); (v) the amount and type of analgesics taken (using frequency tables); and (vi) pain (measured using a visual analog scale [VAS]). The Short Health Scale_HD_ is composed of four questions on how hemorrhoids affect the patient’s daily life, with answers given on a 7-point Likert Scale, and the final total score ranges from 4 to 28 (a higher score indicates worse quality of life) [29]. The Vaizey score measures fecal incontinence, with a score of 0 indicating perfect continence and the maximum score of 24 indicating total incontinence [30]. Bleeding was assessed on a scale of 0–4, where 0 = no bleeding, 1 =  ≤ 1 episode per month, 2 =  ≤ 1 episode per week, 3 = 1–3 episode(s) per week, and 4 = 4 episodes per week. A VAS will be used to measure pain, with a score of 0 indicating no pain and a score of 10 indicating maximum pain.

Assessment of secondary outcomes will take place at baseline, day 10, day 20, and day 40, with the exception of fecal incontinence, which will be assessed only at baseline and day 40.

Patients will also be questioned on day 70 (via telephone) regarding AEs.

AEs occurring from signing of the informed consent form until 30 days after the last administration of study drug or placebo will be recorded and classified according to the latest version of the Medical Dictionary for Regulatory Activities. AEs will be summarized according to system organ class and primary preferred terms, and their severity and relationship to study treatment assessed by the Investigator.

Patient-reported outcomes were assessed via a daily diary, in which patients were encouraged to document the frequency of their main symptoms in order to facilitate clinical evaluation during visits.

### Participant timeline {13}

The participant timeline is shown in Table 1.

**Table 1:** Participant timeline

	**Visits**				
	**T0 (Baseline; Day 0)**	**T1 (Day 10 [± 2 days])**	**T2 (Day 20 [± 2 days])**	**T3 (Day 40 [± 2 days])**	**T4 (Day 70**^**a**^**)**
Written informed consent	X				
Eligibility screen	X				
Demographic data	X				
Objective examination and vital signs	X	X	X	X	
Pathological anamnesis	X				
Previous/concomitant treatments	X	X	X	X	
Pregnancy test	X				
Blood test^b^	X			X	
Proctological examination^c^	X	X	X	X	
Treatment group allocation (i.e., randomization)	X				
Assessment of symptoms (HDSS)	X	X	X	X	
Assessment of HRQoL (SHS-HD)	X	X	X	X	
Assessment of bleeding (Bleeding score)	X	X	X	X	
Assessment of faecal continence (Vaizey score)	X			X	
Assessment of pain (VAS score)	X	X	X	X	
Assessment of AEs		X	X	X	X
Study treatment adherence		X	X	X	
Drug dispensing	X	X	X		

^a^Telephone follow-up

^b^Includes complete blood count, PTT, PT, TT, functional antithrombin III, functional fibrinogen, creatinine, AST, ALT, and CRP

^c^Includes rectal exploration and anoscopy (if applicable)

*AEs*, adverse events; *ALT*, alanine aminotransferase; *AST*, aspartate amino transferase; *CRP*, C-reactive protein; *HDSS*, Hemorrhoidal Disease Symptom Score; *HRQoL*, health-related quality of life; *PT*, prothrombin time; *PTT*, partial thromboplastin time; *SHS-HD*, Short Health Scale for Hemorrhoidal Disease; *TT*, thrombin time; *VAS*, visual analog scale.

### Sample size {14}

The trial aims to enroll a total of 50 patients (25 patients in each treatment group). This sample size is based on an assumed between-group difference in HDSS score of 1.8 points with a standard deviation (SD) of 1.7 (based on results of previous studies [31, 32]), an alpha of 0.05, and a two-tailed *t*-test with 90% power to reject the null hypothesis (i.e., the averages of the two treatment groups are equal) and an expected drop-out rate of approximately 10%.

### Recruitment {15}

Patients will be recruited in hospital when they voluntarily come for a coloproctological examination, until the sample size (*n* = 50) is reached or until October 10, 2024, whichever comes first.

## Assignment of interventions: allocation

### Sequence generation {16a}

The randomization list will be generated by an independent statistician, with stratification according to HD severity (Grade I–III vs external thrombosed hemorrhoids).

### Concealment mechanism {16b}

The randomization list will be sent to the drug packaging manager, then the masked list will be entered into the eCRF system by an information technology manager.

### Implementation {16c}

The randomization list will remain the responsibility of the independent Statistician throughout the duration of the trial. The Statistician will provide envelopes containing individual patient randomization codes to the Investigator, which will remain closed even after the database has been locked.

## Assignment of interventions: Blinding

### Who will be blinded {17a}

Treatment group allocation will remain unknown to patients, investigators, and researchers (i.e., laboratory analysis, data collection, and statistical analysis).

### Procedure for unblinding if needed {17b}

Unblinding of an individual randomization code is permitted in the event of a serious AE (SAE) if it is considered useful for the patient’s safety. Only the Investigator is permitted to open the envelope and the code breach will be clearly indicated in the eCRF and in the envelope itself, which will be re-sealed. The Investigator will inform the trial Monitor immediately that the code has been opened.

## Data collection and management

### Plans for assessment and collection of outcomes {18a}

The CRO, Clinical Trial Consulting, will create and manage the e-CRFs on behalf of the study Sponsor. The Sponsor will then provide this set-format e-CRF to investigators to record the required trial data at each visit (day 0–day 70), with internal controls to help ensure correct data entry. Trial data for each participant will be entered into the e-CRF by the Investigator and collated by the study centers. All safety data (AEs, physical examinations, vital sign measurements and laboratory assessments) will be recorded in patients’ medical records and in the e-CRF. Data cleaning analysis conducted by the Data Manager (an employee of CMV-Stat, Rome, Italy) will identify any inconsistencies to the Investigator for validation (i.e., modification or acceptance).

At the end of the trial, the database will be frozen by the Data Manager and the data sent to the biostatistics unit of CMV-Stat for statistical analysis. All methods used for statistical analyses of data will be listed in detail in the statistical analysis plan (SAP), which will be finalized in Version 1.0 of the SAP, prior to the database lock.

### Plans to promote participant retention and complete follow-up {18b}

Patients will be offered the option to be visited by a clinician from the participating centers at any time, if needed or requested by a patient.

### Data management {19}

Data management and control procedures will be documented in the monitoring plan prior to trial initiation. At the end of the trial, all sets of envelopes will be archived in the trial master file. All essential trial documents will be conserved by the trial centers for ≥ 25 years in accordance with GCP and applicable regulatory requirements.

### Confidentiality {27}

Confidentiality of data will be maintained by the use of coding only to identify patients. Neither the Sponsor nor collaborators may lawfully use patients’ personal data without first obtaining appropriate and specific expressions of consent.

### Plans for collection, laboratory evaluation, and storage of biological specimens for genetic or molecular analysis in this trial/future use {33}

Data and biological samples may be used in future trial and research activities if specific consent to do so has been provided by the individual patients during the written informed consent process. In the event that a patient withdraws from the trial, all stored biological samples permitting patient identification will be destroyed.

## Statistical methods

### Statistical methods for primary and secondary outcomes {20a}

The statistical analysis of data from the trial will be conducted by a statistician from CMV-Stat on behalf of the Sponsor.

All the variables will be analyzed descriptively using means with SDs, medians with interquartile ranges (IQRs), minimum and maximum values, and 95% confidence intervals (where appropriate) for continuous variables, and numbers, percentages, and missing data for categorial variables.

For all trial endpoints, analyses will be performed on each timepoint in absolute terms and as changes from baseline. Efficacy endpoints will be analyzed according to the stratification factor of the presence of uncomplicated HD versus thrombosed hemorrhoids. Efficacy analysis will be applied in the intention-to-treat (ITT) and per protocol (PP) populations and safety/tolerability will be assessed in the safety population. The ITT population is defined as all patients who have taken ≥ 1 dose of study drug/placebo and who have ≥ 1 post-baseline assessment for the primary endpoint. The PP population is defined as all enrolled patients who have data from all observations, met all inclusion/exclusion criteria, not taken any unauthorized drug, and taken ≥ 80% of the study drug/placebo. The safety population is defined as all patients who have signed informed consent and have taken ≥ 1 dose of study drug/placebo.

For the primary endpoint (i.e., change in HDSS from baseline to day 40 in the ITT population), the *t*-test will be applied with a significance level of 5%. If assumptions of normality and homoscedasticity are unmet, a corresponding non-parametric test will be applied.

### Interim analyses {21b}

There are no interim analyses planned.

### Methods for additional analyses (e.g., subgroup analyses) {20b}

There are no subgroup analyses planned. However, a sensitivity analysis may be added to the SAP according to the following subgroups: “patients with grade I, II or III HD” and “external thrombosed hemorrhoids”, if necessary.

### Methods in analysis to handle protocol non-adherence and any statistical methods to handle missing data {20c}

Statistical methods for substitution of missing values will be listed in the SAP, which will be finalized in Version 1.0 of the SAP, before the database lock.

### Plans to give access to the full protocol, participant-level data, and statistical code {31c}

Investigators will permit direct access to and review of all original trial documentation (signed informed consent forms, medical records, outpatient records) to the regulatory authorities, ethics committee, or the Sponsor with the provision that patient identities remain confidential.

Results of the trial will be documented by the trial Sponsor in an integrated clinical–statistical final report in accordance with GCP/International Council for Harmonization (ICH) guidelines.

## Oversight and monitoring

### Composition of the coordinating center and trial steering committee {5d}

The CRO is responsible for clinical monitoring activities as detailed in the monitoring plan drawn up in accordance with European Union GCP Guidelines.

### Composition of the data monitoring committee, its role and reporting structure {21a}

A data monitoring committee will not be utilized for this study. Instead, periodic monitoring visits will be conducted by the CRO. The CRO’s designee will review study data for accuracy, completeness, and consistency by comparing the data to that indicated on the source document during on-site monitoring visits and after transmission to the sponsor. Any discrepancies will be resolved by the investigator or designee as needed.

### Adverse event reporting and harms {22}

All AEs (serious and non-serious) which occur from signing the informed consent until 30 days after the last administration of the study drug will be recorded and described in the patient’s medical records and in the e-CRF. All events meeting the definition of serious will be reported as a SAE, regardless of whether or not they are identified during a protocol-specific assessment/visit. All SAEs must be reported by the Investigator to the Sponsor using the SAE form within 24 h of becoming aware of them; the initial report of a SAE may be reported by email.

Death is considered a SAE regardless of whether the event is expected or not, or considered associated with the study treatments. Any event requiring hospitalization (or prolongation of hospital stay) that occurs during a patient’s participation in a trial must be reported as a SAE, with the exception of hospitalizations for social reasons (in the absence of an AE) or pre-planned surgery/procedures. All SAEs must also be reported to the relevant ethics committee.

### Frequency and plans for auditing trial conduct {23}

Prior to initiating the trial, the trial Monitor will visit each center to clarify practical details of the trial (e.g., review of the protocol; procedures for obtaining informed consent, completion of the e-CRF and reporting of SAEs; management of the trial interventions [handling, storage, etc.] and trial documentation). During the trial, regular monitoring will be scheduled (2 × center visits and 4 × remote) by the trial Monitor to check the completeness and coherence of the data entered in the e-CRF, and compliance with trials procedures. In addition to these monitoring procedures, audits will be carried out by the Sponsor to ensure trial activities have been conducted and data recorded, analyzed, and reported in accordance with the protocol, GCP, standard operating procedures, and Italian regulatory requirements. Also, documents, facilities, and records related to the trial may be inspected by national/international regulatory authorities.

### Plans for communicating important protocol amendments to relevant parties (e.g., trial participants, ethical committees) {25}

Any protocol amendments will be submitted by the Sponsor to the ethics committee and approval documented prior to implementation. If the amendment substantially alters the design of the trial, each patient will be informed and their willingness to continue the trial will be confirmed in writing using an approved information sheet and consent form provided by the Sponsor.

### Dissemination plans {31a}

A copy of the final clinical trial report will be sent to relevant regulatory authorities (e.g., ethics committees), and the trial results may be published by the Sponsor, and/or presented at scientific conferences, after approval by the Scientific Coordinator.

## Discussion

The double-blind, randomized, controlled CHORMES trial is designed to investigate the efficacy and safety of mesoglycan versus placebo in patients with HD (Grade I–III or thrombosed external hemorrhoids). The effects of treatment on symptoms of HD and the impact on patient HRQoL will be evaluated. Analgesic use during treatment and pain will also be examined.

While an active comparator has not been included, the results derived from the study will allow clinicians to assess, albeit indirectly, the relative efficacy and safety of mesoglycan versus other available bioflavonoids for the treatment of acute HD. This includes, for example, a mixture of flavonoids investigated by Giannini and colleagues [31], and a micronized purified flavonoid fraction [33].

The key clinical endpoints of the current trial will be assessed using validated rating scales/scores (e.g., the HDSS [primary endpoint], the Short Health Scale_HD_, and the Vaizey score) in an attempt to make (indirect) comparisons versus trials of other flavonoid products for acute HD more relevant, although some authors have noted there remains a lack of standardized outcomes used in HD research [34], as well as interobserver variability in HD grading (as previously mentioned) [9].

A strength of the trial is its prospective, randomized, double-blind design, and it is the first study to evaluate the efficacy and safety of mesoglycan for acute HD in this standardized manner. It is the first standardized mesoglycan trial in patients with Grade I–III HD or external thrombosed hemorrhoids. As such, the trial results should provide good quality evidence to support its use in this patient population and will provide an update on the only placebo-controlled studies of mesoglycan, both of which were conducted over 35 years ago [27, 28]. Further, to the best of our knowledge, it is the first standardized mesoglycan trial in patients with Grade I–III HD or external thrombosed hemorrhoids to include a placebo control. As such, the trial results should provide good-quality evidence to support its use in this patient population and will provide an update to the existing literature. The two study centers from which patients will be recruited serve a high number of patients with HD across a wide range of HD severities, which implies that a good selection of patients will likely be enrolled into the study. However, the trial does have some limitations, in particular the relatively small anticipated sample size (*n* = 50), and that the use of concomitant medications such as analgesics and stool softeners will be allowed (although this will be recorded to allow for comparison between trial arms). Additionally, it is possible that patients with more severe disease are likely to be treated at the two study centers, so it is possible the patient population may be skewed towards patients with more serious pathology, although randomization procedures should reduce this risk*.*

## Trial status

The trial is currently operating under protocol Version 2.0, dated April 18, 2023. The first participant was recruited on 11 December 2023, and it is estimated that recruitment will be completed by October 2024 and the trial completed by December 2024.

## Supplementary Information

Supplementary file 1.

## Data Availability

Data sharing is not applicable to this article as it reports the protocol of the CHORMES trial; however, once the trial is completed, the datasets used and/or analyzed during the study will be available from the corresponding author on reasonable request.

## Abbreviations

AE(s): Adverse event(s)
CHORMES: ACute HaemORrhoids treatment with MESoglycan trial
CRO: Contract Research Organisation
e-CRF: Electronic case report form
GAGs: Glycosaminoglycans
GCP: Good Clinical Practice
HD: Hemorrhoidal disease
HDSS: Hemorrhoidal Disease Symptom Score
HRQoL: Health-related quality of life
IHC: International Council for Harmonisation
IQR(s): Interquartile range(s)
ITT: Intention-to-treat
PP: Per protocol
SAE: Serious adverse event
SAP: Statistical analysis plan
SD: Standard deviation
Short Health Scale_HD_: Short Health Scale for Hemorrhoidal Disease
