# Supplementary material for: Use of mesoglycan in the acute phase of hemorrhoidal disease (the CHORMES study): study protocol for a double-blind, randomized controlled trial
Source: Trials. 2024 Dec 2;25:807. doi: 10.1186/s13063-024-08648-y (PMC11613797; doi:10.1186/s13063-024-08648-y)
Supplement: Supplementary file 1 [file 13063_2024_8648_MOESM1_ESM.pdf]

LETTERHEAD OF THE INSTITUTION
HOSTING THE TRIAL

INFORMATION SHEET AND INFORMED CONSENT FORM FOR PATIENT PARTICIPATION IN A CLINICAL TRIAL

**Official title of the trial** (in English): **Randomized Double-blind Controlled Clinical Trial: the Use of Mesoglycan vs Placebo in the Acute Phase of Hemorrhoidal Disease**
**Official title of the trial in terms more understandable to the patient** (use common, non-technical terms: for example, make it explicit that the molecule being tested is a new drug to lower blood pressure, prevent a heart attack, etc.): **Study on the use of Mesoglycan (Prisma®) in the acute phase of Haemorrhoidal Disease.**
**Facility-context in which the trial will take place**<br>Azienda Ospedialiera-Universitaria Policlinico di Bari - Outpatient Department of Coloproctology Surgery "M. Rubino"
**Coordinating centre** (if different from the facility where the trial will take place) **and trial coordinator**<br>Coordinating Centre Azienda Ospedialiera-Universitaria Policlinico di Bari - Outpatient Department of Coloproctology Surgery "M. Rubino"<br>Coordinator of the trial Dr Marcella Rinaldi
**Principal investigator** (specify local investigator in charge)<br>Name Dr Marcella Rinaldi<br>Affiliation Azienda Ospedialiera-Universitaria Policlinico di Bari - Outpatient Clinic of Coloproctology Surgery "M. Rubino"
**Sponsor/Funding body:** NEOPHARMED GENTILI S.p.A., Via S. Giuseppe Cottolengo, 15 - Milan
**Ethics Committee**<br>Interregional Ethics Committee – Policlinico di Bari - P.zza G. Cesare n. 11, Bari- 70124

This document consists of the following sections:

A. INTRODUCTION
B. INFORMATION SECTION. SUMMARY OF THE TRIAL: KEY INFORMATION
C. INFORMATION SECTION. FURTHER DETAILS
D. DECLARATION OF CONSENT SECTION

ATTACHMENTS
LETTER FOR THE GP

Dear Sir/Madam, The information in the following information sheet is very detailed. We ask that you agree to participate in the trial ONLY after you have read this information sheet carefully and have had an EXHAUSTIVE DISCUSSION with a member of the trial team, who must give you ALL THE TIME YOU NEED to fully understand what is being proposed to you.

A. INTRODUCTION

Dear Sir/Madam,
We are inviting you to take part in the clinical trial described below.
It is your right to be informed about the purpose and characteristics of the trial so that you can make an informed and free decision whether or not to take part.
The aim of this document is to inform you about the nature of the trial, its intended purpose and what your participation will entail, including your rights and responsibilities.
Please read the following carefully. The researchers involved in this project, listed at the beginning of this document, shall be happy to answer your questions. No question is trivial so don't be afraid to ask it!
As well as with us, you can discuss the proposal in this document with your GP, family members and other people you trust. Take as much time as you need to decide. You may take home an unsigned copy of this document to think about or discuss with others before making a decision.
If you decide not to participate in the trial, you will still receive the best possible care for patients with your condition/disease.
Your refusal to take part will in no way be interpreted as a lack of trust.

If you are unable to sign the informed consent, consent may be given and recorded by appropriate alternative means, such as audio or video recordings before at least one impartial witness.

Once you have read this form, had any questions answered and potentially decided to participate in the trial, you will be asked to sign a consent form, a copy of which will be given to you.

The Principal Investigator

B. INFORMATION SECTION.

GENERAL SUMMARY OF THE TRIAL: KEY INFORMATION

The aim of this section is to briefly present the key aspects of the trial we are inviting you to join. The following sections will provide more details in order to give you the opportunity to grant or not grant your fully informed consent to take part in the trial.

Why am I being asked to participate in this trial?

We are asking you to participate in a clinical trial funded by NEOPHARMED GENTILI S.p.A. because you have acute haemorrhoidal disease and we want to see whether the trial therapy may be more effective than those currently used.
You are being asked to participate in this trial because you have certain clinical characteristics that will be further specified in Section C.

What are the objectives of the trial? How many centres and patients will take part?

The trial is being conducted with the aim of improving symptoms and reducing the impact of haemorrhoidal disease on quality of life. The medication is already being used for other diseases and the aim of the trial we are inviting you to participate in is to see if it is also effective in your disease and to define the best dosage.
Other secondary objectives of the study are to reduce bleeding and the amount and type of pain medication taken. The trial is planned to take place in 2 Italian centres and 50 patients will be included.

What is the routine care approach for the treatment of haemorrhoidal disease?

Grade I-III internal haemorrhoids are effectively treated with diet and lifestyle changes and medical therapy and/or, if unsuccessful, outpatient procedures such as elastic ligation and sclerotherapy (a medical procedure by which blood vessel malformations are treated).

Am I free to decide whether or not to participate?

You can freely choose whether or not to participate in the trial. Even after agreeing, you can change your mind at any time.

If I decide not to give my consent to participate in the trial, what choices do I have?

If you decide not to join the trial, you can still be monitored by the clinical centre treating you and will be treated using the best approved (non-investigational) therapeutic methods for your disease.
In addition, you may participate in another trial that may be ongoing.

What happens if I decide to participate in the trial?

If you decide to participate in the trial, you will be randomly assigned to either mesoglycan or placebo, which you will have to take for up to 40 days or until treatment is stopped early.
During this period, you will be asked to visit the centre that is monitoring you for 4 follow-up visits. The full schedule of visits and examinations planned during the trial is provided in the next section “What examinations, tests and procedures are planned during the trial?”
If you agree to participate, you will take mesoglycan 50 mg or a placebo, according to the following treatment schedule:

- 4 capsules/day orally for the first 5 days;
- 2 capsules/day orally for the next 35 days.

You are to take the medication twice a day, after breakfast and after dinner.
If you completed the trial and benefited from the new treatment, you will not be able to continue taking it. In the event that further research with that particular product is abandoned for your condition, your GP will re-evaluate your treatment options.
Before taking part in the trial, the doctor verified that you had the required characteristics to take part. Invasive procedures (proctological visit with rectal examination and anoscopy, where possible), explained in more detail below, shall be scheduled during the trial.

What are the risks and benefits if I participate in the trial?
Taking part in this trial may entail both risks and benefits. It is important to evaluate them carefully before making a decision.

Expected benefits
If you are assigned to treatment with mesoglycan, the potential benefit from participating in the study will be the reduction of symptoms or potential remission of symptoms due to treatment with mesoglycan.
If you are assigned to placebo, you will receive treatment according to the current best standard of care and benefit from timely and accurate clinical surveillance because of your participation in the study.
By joining the trial, you will have the opportunity to be treated with a medication that may be better than those currently on the market, and you will also contribute to the development of new drugs for your disease. In the future, you and other patients with your disease may benefit.

Risks
The risks of participating in the study are the occurrence of any undesirable side effects due to mesoglycan treatment. If you decide to take part in this trial, there is a risk that the investigational medication/treatment will be less effective than the standard medication/treatment. There is also a risk that more serious adverse reactions may occur with the investigational treatment than with standard treatment.
Clinical experience of mesoglycan use showed a low incidence of adverse reactions (mainly non-serious abdominal pain and dyspepsia and non-serious skin reactions). In randomised, placebo-controlled clinical studies, the risk profile of mesoglycan was not dissimilar to that of the placebo. Of particular note was the rarity of clinically relevant haemorrhagic adverse events.
You will be closely monitored for each of these reactions. However, not all adverse reactions that may occur are known.
The known significant adverse reactions of the investigational treatment are mainly non-serious upper gastrointestinal tract disorders and non-serious skin reactions.
Any treatment taken for conditions unrelated to the disease being studied and considered necessary for the patient’s well-being and, moreover, which does not interfere with the study medication, may be administered at the Investigator’s and GP’s discretion.
The use of painkillers as analgesic therapy as needed and faecal emollients is also permitted.

Is consent final? Can I decide to withdraw from the clinical trial (voluntary drop-out)?
You can decide to withdraw from the trial at any time and for any reason, without having to justify your decision.

Should you decide to withdraw from the trial, no further data about you can be collected, and any biological samples taken and stored in a form that allows you to be identified will be destroyed. However, any data already collected may still be used, unaltered, to determine the results of the research.
If you decide you no longer wish to take part, please let one of the investigating doctors know as soon as possible, as it is important to discontinue the treatment safely. The doctor may consider that a final follow-up visit/examination is appropriate.
The doctor will keep you informed of any changes in the trial that may affect your willingness to participate.

Are there any reasons why the trial could be terminated not of my own volition (early termination)?
Yes, the investigating doctor may decide to stop your participation in the trial if:
- Your health condition should change and participating in the trial would be potentially harmful.
- New information becomes available and the trial was no longer in your best interest.
- You do not follow the agreed rules for participation in the trial.
- For women: you fall pregnant during the trial.
- The trial is stopped by the competent authorities or the sponsor.

C. INFORMATION SECTION. FURTHER DETAILS

What is the purpose of the trial?

The purpose of this trial is to evaluate efficacy and safety of mesoglycan, compared with placebo, in resolving symptoms in acute haemorrhoidal disease. In total, 50 people will participate in the trial.

What patient groups are being compared? What is the intervention being tested?

The study population will consist of patients with grade I-III haemorrhoidal disease and external thrombosed haemorrhoids who have provided written informed consent and meet the eligibility criteria below. Patients will be randomly enrolled in either the investigational medication arm (mesoglycan 50 mg) or the placebo arm in a 1:1 ratio.

Patients enrolled in the investigational arm will take mesoglycan 50 mg or a placebo, according to the following treatment schedule:

- 4 capsules/day orally for the first 5 days;
- 2 capsules/day orally for the next 35 days.

The treatment should be taken twice a day, after breakfast and after dinner.

Those individuals who meet the following inclusion criteria and none of the exclusion criteria below will be eligible to participate in the study.

Inclusion criteria

1. Patients of both genders aged between 18 and 75 years.
2. Diagnosis of grade I-III haemorrhoidal disease or diagnosis of external thrombosed haemorrhoids.
3. Negative pregnancy test for female patients of childbearing age, who must agree to use an effective barrier contraceptive method for the entire duration of the study.
4. Patients able to understand and sign the informed consent form.
5. Patients who sign the informed consent form.

Exclusion criteria

1. Patients with a remote medical history of coagulopathy, heart disease, anal or colorectal neoplasia, chronic inflammatory bowel disease, previous perianal surgery or other acute-phase proctological diseases.
2. Patients currently undergoing or having completed radiotherapy within the past 3 years.
3. Patients being treated with anticoagulant and/or anti-platelet therapy.
4. Any contraindication to treatment or known intolerance to mesoglycan.
5. Patients with galactose intolerance, lapp-lactase deficiency or glucose-galactose malabsorption.
6. Women who are pregnant or breast-feeding, or of childbearing age who are not using an effective method of birth control. Women of childbearing age must have a negative pregnancy test at enrolment. Post-menopausal women must have had amenorrhoea (no menstrual periods) for at least 12 months to be considered potentially infertile.
7. Any condition that, in the opinion of the investigator, might put the patient at risk from participating in this study.
8. Patients taking part in other clinical trials.

What examinations, tests and procedures are planned during the trial?

This study involves 4 visits to the centre that is monitoring you.
During the first visit, some demographical data (age, gender, ethnicity) will be collected, a physical examination will be performed and a medical history taken. Any other (concomitant) therapies you are taking will be checked. You will be asked to take a pregnancy test if you are of childbearing age. A blood sample will be taken and a proctological visit (rectal examination and anoscopy, where possible) will be performed. Some rating scales will be applied and finally you will be given the medication.
The second visit will take place after 10 days. During the visit, a physical examination will be performed and your vital signs and any concomitant therapies will be checked, the proctological visit will be repeated, some rating scales will be applied and finally you will be given the medication.
The third visit will take place after another 10 days. During the visit, a physical examination will be performed and your vital signs and any therapies will be checked, the proctological visit will be repeated, some rating scales will be applied and finally you will be given the medication.
The last visit will be held after a further 20 days. During the visit, a physical examination will be performed and your vital signs and any concomitant therapies will be checked, another blood sample will be taken, the proctological visit will be performed and some rating scales will be applied.
For each and every invasive examination or intervention performed as part of the trial, a specific consent will be collected.
Below is a table detailing the study and procedures performed during each visit.

**Procedures**	**Visit T0**	**Visit T1**	**Visit T2**	**Visit T3**
	**Baseline** (Day 0)	**10 days** (± 2 days)	**20 days** (± 2 days)	**40 days** (± 2 days)
Written Informed Consent	✓			
Inclusion/Exclusion Criteria	✓			
Randomisation	✓			
Demographic data	✓			
Medical History	✓			
Physical Examination and Vital Signs	✓	✓	✓	✓
Concomitant and prior therapies	✓	✓	✓	✓
Blood sampling for safety assessment*	✓			✓
Pregnancy test (kit)	✓			
Proctological visit (rectal examination and anoscopy, when applicable)	✓	✓	✓	✓
HDSS (Haemorrhoidal Disease Symptom Score)	✓	✓	✓	✓
SHS-HD (Short Health Scale for Haemorrhoidal Disease)	✓	✓	✓	✓
Bleeding Score	✓	✓	✓	✓
Vaizey Score (Assessment of faecal continence)	✓			✓
Visual Analogue Scale (VAS)	✓	✓	✓	✓
Dispensation of medication	✓	✓	✓	
Accounting of medication		✓	✓	✓
Adverse Events		✓	✓	✓

Is it useful/necessary to inform my GP?
If you decide to participate in the trial, it is important to inform your GP. To this end, we have prepared (or will give you) a letter that you can give to your GP explaining the procedures of the trial.

What will my commitment be and what responsibilities will I have if I decide to participate?
Should you decide to participate, you will be asked to:

- Scrupulously observe the instructions and requests of the trial medical staff and ensure you attend all appointments.
- Inform the doctor monitoring the trial:
    - of all medications you are taking, including unconventional medicines,
    - of any side effects arising during the course of the trial,
    - of any visit to, or hospitalisation in, facilities other than the trial centre,
    - of ongoing or past participation in other clinical trials.

- not fall pregnant or breastfeed during the trial.
- not father a child during the trial.
- inform your doctor promptly if you or your partner plan to become pregnant during the trial or within 30 days after the last dose of the trial medication.

Please remember that the treatment provided in the trial could harm a possible foetus. You are therefore expected to take a pregnancy test before the trial and subsequently commit to not fall pregnant. If you agree to participate in this trial, you must use a safe method of contraception during the trial period and for 30 days after the last dose of the medication. You should discuss the best contraceptive method for your circumstances with the trial doctor.

Will I incur costs for participating in the trial? Will I be reimbursed for any expenses? Will I receive compensation?
You will not incur any costs for participating in the trial as these are fully covered by the Sponsor.
You will also not be financially compensated for taking part in the trial.

What happens if I am harmed as a result of participating in the trial?
Participation in a clinical trial may involve drawbacks and risks that cannot be determined in advance. For this reason, the clinical trial provides insurance coverage to protect your participation.
In compliance with applicable laws, insurance shall be arranged to cover any harm incurred as a result of taking part in the trial, for its entire duration, covering the civil liability of the investigator and the sponsor.
STATE THE INSURANCE COMPANY, POLICY NUMBER, MAXIMUM AMOUNT PER PARTICIPANT AND THE AGGREGATE MAXIMUM AMOUNT, the details of which are attached.
It should be noted that, according to the Italian Ministerial Decree of 14 July 2009, the insurance policy does not cover a value in excess of the maximum amount and applies only to harm for which the claim has been filed no later than the period stipulated in the policy (ENTER THE NUMBER OF MONTHS). However, this limitation does not affect your right to obtain compensation from the party responsible for any harm (to protect the trial subject).

How will my health data, including identifying data, be processed and who will have access to them during the trial?
Your data, particularly personal and health data and only to the extent that they are indispensable to the objective of the trial and for pharmacovigilance purposes, will be processed in accordance with EU Regulation 2016/679, known as GDPR (General Data Protection Regulation) and Italian Legislative Decree No. 196 of 30 June 2003, as amended by Italian Legislative Decree No. 101 of 10 August 2018.

In practical terms, your name will not appear on documents or in the study files, your data will be pseudonymised, and a "unique subject identification code" will be used and assigned to you at the beginning of the study. This "unique subject identification code" will be unique and will be the only identifiable information on the study documents.
Sometimes, during a study, it is necessary to verify that the study is being conducted properly at the relevant clinical centre. To do this, in addition to medical personnel, people representing the study sponsor, the Sponsor itself, ethics committees or regulatory authorities may sometimes need to review your medical records at the clinical centre in order to verify that the information has been entered correctly in the study documents and to ensure that the clinical centre has followed the study guidelines and procedures. In order to perform these checks, the people named above may need direct access to your relevant medical records. If this is necessary, it will be done under the direct supervision of the trial team, and all information reviewed by these people will remain confidential.
The results of the study will be published in medical journals and/or presented at medical meetings or conferences. Only anonymised results will be published, and no patient-identifying information will be included in any publication.
During the study, information will be collected from your medical records, which will be entered into a secure computerised database. This will be done by trial staff members and the information will be pseudonymised. Your name will not be used in the database. This means that the “unique subject identification code” assigned to you at the beginning of the study will be used to enter all data into the study database. The Investigator is responsible for collecting the data for the study itself.
Your clinical data collected for the purpose of the trial, as well as the results of examinations performed, will be retained for the time required by regulations and then destroyed. It will not be destroyed only in the following cases: a) it can no longer be traced back to your identity because it was anonymised during the trial itself; b) if you give your specific informed consent.
Prior to transferring any personal data to a third country or international organisation, the assessments set forth by Chapter V of the GDPR will be carried out; data will only be transferred if the established requirements are met.
Please be aware that the Sponsor may use your data and biological samples in future study and research activities, including making use of the external parties who collaborated with them to conduct the trial, if you consent.
You may exercise all the rights recognised by the GDPR, including the right to erasure of data, unless this is precluded by regulations or legal documents, or current data protection legislation authorises its use in the overriding interest of research.
This point does not constitute information pursuant to Article 13, GDPR, proposed separately to the person concerned (data subject) and to which express reference is made.

How will I have access to the results of the trial?
Once the trial is completed and all the resulting data have been collected, they will be analysed to draw conclusions. The investigators and the sponsor agree to make them available to the scientific community.
Regulations provide for participant access to the results of the trial. Therefore, you may ask the investigator to inform you of the trial’s overall results.

Has the trial been approved by the Ethics Committee?

The trial protocol proposed to you has been reviewed and approved by the General Hospital of Bari's Interregional Ethics Committee. Among other things, the Ethics Committee verified that the trial complied with the Guideline for Good Clinical Practice and the ethical principles expressed in the Declaration of Helsinki and that your safety, rights and well-being have been protected.

Who can I contact for more information about the clinical trial I am invited to take part in?

Dr..........................
e-mail: ....
Tel.: +39 ....

If I join the trial, who will I be able to contact should I need to?
For any concerns or unscheduled or unplanned events during the course of the trial (concerns about the current treatment, side effects, decision to leave the trial, etc.), you may contact:
Dr..........................
e-mail: ....
Tel.: +39 ....

Should you deem it appropriate to report events or facts pertaining to the trial in which you have participated to subjects not directly involved in the trial itself, you may contact the Ethics Committee that approved the trial (Interregional Ethics Committee – General Hospital of Bari), the Health Management of the trial centre (General Hospital of Bari) or the competent authority (AIFA).

_________________________________ ___/___/______ __________
Full name of the doctor who provided the information sheet Date Time

____
Signature

Attachments
- Insurance policy
- Personal data processing consent form

Additional documents:
- Letter for the GP

D. DECLARATION OF CONSENT SECTION

(Notes: 1 copy for the participant, 1 copy for the trial coordinator)

Title of the trial: Randomized Double-blind Controlled Clinical Trial: the Use of Mesoglycan vs Placebo in the Acute Phase of Hemorrhoidal Disease.

Protocol code, version and date:

Trial **sponsor**/funding body: NEOPHARMED GENTILI S.p.A.

Principal Investigator Prof. Marcella Rinaldi - Azienda Ospedialiera-Universitaria Policlinico di Bari - Outpatient Clinic of Coloproctology Surgery "M. Rubino"

I the undersigned

____

born in ____on

___/___/______

DECLARE

- ▢ that I have received from Dr ______________________________________ comprehensive explanations regarding the request to participate in the research in question, as stated in the information section, forming part of this consent, a copy of which was given to me on ____________ at ______________ (enter date and time of receipt);
- ▢ that the nature, purposes, procedures, expected benefits, possible risks and drawbacks, and alternative treatment methods to the proposed clinical trial were clearly explained to me and that I understood them;
- ▢ that I had the opportunity to ask the study investigator any questions and received satisfactory answers;
- ▢ that I had sufficient time to reflect on the information received;
- ▢ that I had sufficient time to discuss it with third parties;
- ▢ that I have been informed that the trial protocol and all the forms used have received the favourable opinion of the relevant Ethics Committee;
- ▢ that I am aware that the research may be terminated at any time at the discretion of the trial coordinator;
- ▢ that I have been informed that I will be made aware of any new data that may compromise the safety of the research and that, for any problems or further questions, I can contact the doctors treating me;
- ▢ that for the best protection of my health, I am aware of the importance (and my responsibility) of informing my GP of the trial I am agreeing to take part in. I am aware of the importance of providing all information (medication, side effects, etc.) about me to the investigator;
- ▢ that I have been informed that the results of the study will be made known to the scientific community, protecting my identity in accordance with current privacy regulations;

▢ that I am aware that any choice expressed in this consent form may be revoked at any time and without justification and that, in such an event, no further data concerning me will be collected and any biological samples that may have been collected and stored in a form that allows me to be identified will be destroyed; without prejudice, however, any data already collected may still be used, unaltered, to determine the results of the research;
▢ that I have received a copy of this consent form.

I therefore DECLARE

▢ to want	▢ to NOT want	to participate in the trial
▢ to want	▢ to NOT want	to be informed of any unexpected news pertaining to my current or future health that may incidentally arise from the investigations involved in the trial, including genetic investigations, when this may result in possible benefits *(this applies only if you have decided to participate in the trial*)
▢ to want	▢ to NOT want	to be informed of unexpected news about my current or future health only when it may be useful for my health care or to enable me to make informed reproductive choices *(this applies only if you have decided to participate in the trial*)
▢ to want	▢ to NOT want	to be contacted again after the end of the trial to provide information about my health status *(this applies only for contacts not scheduled as follow-up by the study protocol; this applies only if you have decided to participate in the trial*)

If applicable:

▢ to agree	▢ to NOT agree	to the use of contraceptive methods

_______________________________ ___/___/______ _________
Full name of adult patient Date Time

____
Signature

_______________________________ ___/___/______ _________
Full name of
legally designated representative
or Guardian (if applicable)
Date Time

____
Signature

▢ I authorise ▢ I do NOT authorise

the Sponsor to use my data and biological samples in future study and research activities, including making use of the external parties who collaborated with the Sponsor in conducting the trial.

_______________________________ ___/___/______ _________
Full name of adult patient Date Time

____
Signature

_______________________________ ___/___/______ _________
Full name of
legally designated representative
or Guardian (if applicable)
Date Time

____
Signature

STATEMENT FROM THE DOCTOR WHO COLLECTED THE CONSENT

____
(Patient's name, place and date of birth)

Title of the trial: Randomized Double-blind Controlled Clinical Trial: the Use of Mesoglycan vs Placebo in the Acute Phase of Hemorrhoidal Disease.

Protocol code, version and date:

Trial **sponsor**/funding body: NEOPHARMED GENTILI S.p.A.

Principal Investigator Dr Marcella Rinaldi - Azienda Ospedialiera-Universitaria Policlinico di Bari - Outpatient Clinic of Coloproctology Surgery "M. Rubino"

I, the undersigned Prof./Dr ____ (first and last name), in my capacity as Principal Investigator (or delegate of the Principal Investigator)

DECLARE

that the patient voluntarily agreed to take part in the trial

I further declare:

- ▢ to have provided the patient with comprehensive explanations regarding the purpose of the trial, the procedures, the possible risks and benefits, and its possible alternatives;
- ▢ to have verified that the patient has sufficiently understood the information provided to him/her;
- ▢ to have given the patient the necessary time and opportunity to ask questions about the trial;
- ▢ to have clearly explained the possibility of withdrawing from the trial at any time or changing the choices made;
- ▢ to have not exercised any coercion or undue influence in requesting this consent;
- ▢ to have provided the patient with information on how the results of the trial will be made known to him/her.

_____________________________ ___/___/______ _________

Full name of the doctor who provided the information and obtained consent — Date — Time

____

Signature

This form is an integral part of, and should be kept together with, the informed consent information form
